# NTRK Fusion in Non-Small Cell Lung Cancer: Diagnosis, Therapy, and TRK Inhibitor Resistance

**DOI:** 10.3389/fonc.2022.864666

**Published:** 2022-03-17

**Authors:** Fangfang Liu, Yuxuan Wei, Huan Zhang, Jizong Jiang, Peng Zhang, Qian Chu

**Affiliations:** ^1^ Department of Oncology, Tongji Hospital of Tongji Medical College, Huazhong University of Science and Technology, Wuhan, China; ^2^ The Second Clinical College of Tongji Medical College, Huazhong University of Science and Technology, Wuhan, China

**Keywords:** non-small cell lung cancer, NTRK fusion, diagnosis, TRK inhibitor, resistance

## Abstract

Neurotrophic tropomyosin receptor kinase (NTRK) gene fusion has been identified as an oncogenic driver of various solid tumors, and it is rare in non-smalll cell lung cancer (NSCLC) with a frequency of approximately less than 1%. Next-generation sequencing (NGS) is of priority for detecting NTRK fusions, especially RNA-based NGS. Currently, the tropomyosin receptor kinase (TRK) inhibitors have shown promising efficacy and well tolerance in patients with NTRK fusion-positive solid tumors, regardless of tumor histology. The first-generation TRK inhibitors (larotrectinib and entrectinib) are recommended as the first-line treatment for locally advanced or metastatic NSCLC patients with positive NTRK fusion. However, TRK inhibitor resistance can eventually occur due to on-target or off-target mechanisms. Further studies are under investigation to overcome resistance and improve survival. Interestingly, NTRK fusion might be the mechanism of resistance to epidermal growth factor receptor (EGFR)-tyrosine kinase inhibitors (TKI) in NSCLC patients with EGFR mutation. Regarding immunotherapy, the efficacy of immune checkpoint inhibitors in NSCLC patients harboring NTRK fusion has yet to be well described. In this review, we elucidate the function of NTRK genes, summarize the diagnostic techniques for NTRK fusions, and present clinical data for TRK inhibitors; we also discuss potential mechanisms of resistance to TRK inhibitors.

## Introduction

Lung cancer is the second most common cancer worldwide but remains the leading cause of cancer-related death according to the latest cancer statistics, accounting for almost one-quarter of all cancer deaths (1). In recent years, targeted therapy with small molecular tyrosine kinase inhibitors targeting the EGFR/ALK/ROS1, and immunotherapy blocking immune checkpoints have been approved to treat patients with nonsmall cell lung cancer (NSCLC), and of note, the overall survival and quality-of-life have been drastically improved (2, 3). In addition, the diagnosis and therapy of gene fusions including ALK and ROS1 were revolutionary for TKI therapy in NSCLC, demonstrating remarkable antitumor effects (4–6). Therefore, the novel gene fusion of neurotrophic tropomyosin receptor kinase (NTRK) family has gained popularity recently for clinical research. NTRK genes involving NTRK1, NTRK2 and NTRK3, encode the proteins of tropomyosin receptor kinase (TRK) family TRKA, TRKB and TRKC respectively, which are transmembrane receptor tyrosine kinases. NTRK gene fusions including NTRK1, NTRK2, and NTRK3 fusions are identified as oncogenic drivers in various types of tumors (7). The detection of NTRK gene fusion is recommended by the National Comprehensive Cancer Network (NCCN) clinical practice guidelines, and the TRK inhibitors (larotrectinib and entrectinib) are preferred as the first-line treatment for locally advanced or metastatic patients with NTRK-fusion-positive NSCLC (8). In this review, we describe the molecular biology and functions of NTRK gene. We also summarize the diagnostic techniques of NTRK gene fusions and the clinical data of TRK inhibitors, further discuss the therapeutic strategies and potential mechanisms of TRK inhibitor resistance.

## NTRK Gene and NTRK Fusion

### NTRK Genes and TRK Receptors

**Figure 1:**
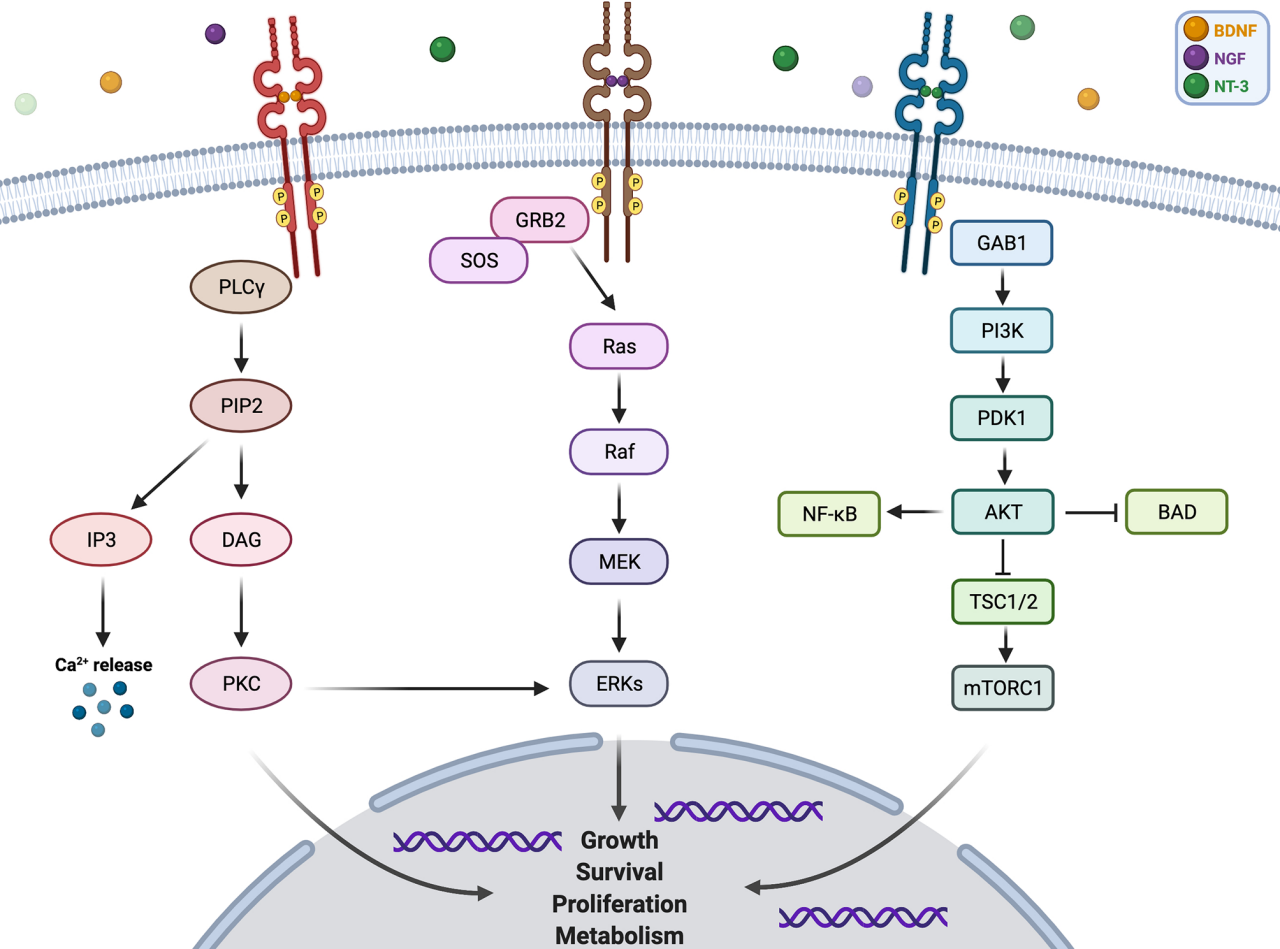
Pathway and function of NTRK gene.

NTRK1 gene is localized on chromosome 1q21–q22 (9), and its encoding protein TRKA binds to the nerve growth factor (NGF) to induce the tyrosine phosphorylation and tyrosine kinase activity of TRKA (10). NTRK2 gene is located on chromosome 9q22.1 (11), and the protein TRKB specifically binds to brain-derived neurotrophic factor (BDNF) (12). Moreover, NTRK3 gene is located on chromosome 15q25 (13), and the TRKC selectively binds to neurotrophin 3 (NT-3) (14). Furthermore, the NT-3 binds to all three TRK receptors, and the interaction between NT-3 and TRKC elicits a more efficient biological response than that with TRKA or TRKB (14, 15). Additionally, each of the TRK proteins is composed of an extracellular domain, a transmembrane region, and an intracellular region containing the tyrosine kinase domain (16). The bind of ligands and TRK receptors causes TRK receptor dimerization, which activates multiple intracellular signaling pathways involving phospholipase C-γ (PLCγ), PI3 kinase (PI3K), and mitogen-activated protein kinase (MAPK) pathways (17). These three pathways play important and different roles in cell functioning. MAPK pathway is involved in cell growth and proliferation, while PLCγ pathway regulates neuronal differentiation, survival, and metabolism. PI3K pathway is responsible for metabolism survival and apoptosis prevention (18). There are crosstalks between these signaling pathways to coregulate biological functions of NTRK genes, and the proper activation of TRK receptors is critical to nervous system development and cell survival (**Figure 1**).

### NTRK Fusion

Gene fusions are resulted from genomic rearrangements, such as chromosomal inversions, interstitial deletions, duplications, or translocations, promoting the development and progression of cancer (19). As for the NTRK gene fusions, the 3’ sequences of NTRK gene is fused to the 5’ sequence of a fusion partner gene, which is a typical genetic structure of the oncogenic fusion (20). The resultant novel fusion oncogene is aberrantly expressed, and causes ligand-independent activation of the kinase domain, which is also called constitutive activation. The constitutive activation is generally the result of the 5’ fusion partner gene which contains sequences encoding dimerization domains (19). Thus, it leads to persistent activation of downstream signaling pathways which is essential to tumor maintenance. The NTRK gene fusion TPM3-NTRK1 was initially discovered in colorectal cancer in 1986 (21). NTRK gene fusions were then discovered as oncogenic drivers of various adult and pediatric tumors. In a large-scale study, NTRK fusions with 88 unique fusion partners were identified in 134 histological subtypes among 45 types of cancers (7). However, the frequencies of NTRK gene fusions vary by cancer types. For example, ETV6-NTRK3 fusion is highly enriched in patients with cellular congenital mesoblastic nephroma, congenital fibrosarcoma, and secretory breast carcinoma (22–24), indicating a link between NTRK gene fusion and certain types of cancer histology. A case report showed that a patient initially diagnosed with salivary acinic cell carcinoma was finally reclassified as mammary analog secretory carcinoma after next-generation sequencing (NGS) results, suggesting an ETV6-NTRK3 fusion (25). Additionally, NTRK gene fusions are less frequent in NSCLC. Up to now, multiple NTRK fusion partners have been reported gradually in NSCLC. Vaishnavi et al. described two NTRK gene fusions in lung cancer, MPRIP-NTRK1 and CD74-NTRK1, which result in constitutive TRKA kinase activity and are oncogenic (26). Other NTRK1 fusion partners like SQSTM1, TPR, IRF2BP2, BCL9, LMNA and PHF20 were also detected in NSCLC (27, 28). TPM3 was the most common NTRK1 fusion variant, and TPM3-NTRK1 was reported as a resistance mechanism to both first-generation and third-generation EGFR-TKIs in NSCLC patients (28). Additionally, ETV6 and SQSTM1 were common fusion partners identified for NTRK3 in NSCLC (27).

## Clinical Characteristics of NTRK Fusions

### Frequency and Clinical Characteristics of NTRK Fusions

NTRK fusions exist in various adult and pediatric malignancies, though it is a rare gene alteration with an overall frequency of less than 1% (7, 29–31). The overall prevalence of NTRK fusion was 0.27%, where 31 cases were fusion positive from tissue samples of 11,502 patients (29). In addition, in a study with 26,000 patients, 76 cases were identified with NTRK fusions, suggesting an overall prevalence of 0.28% (30). Evidence from a large real-world population showed that the overall prevalence of NTRK fusion was 0.30% among 45 cancers types, and it varied by age with a higher prevalence in pediatric patients (1.34%) than adults (0.28%), especially in children <5 years (2.28%) (7). Consistently, a recent research showed that pediatric tumors had a higher frequency of NTRK fusions and a broader panel of fusion partners than adult tumors (32). Yet in another study, the frequencies of NTRK fusions assessed from 13,467 samples were 0.34% in pediatric tumors and 0.31% in adult tumors (31). More relevant data are required for confirmed results. Additionally, the frequency of NTRK fusions distinctly varied by cancer type, where rare cancer types such as salivary carcinoma and thyroid cancer had a higher occurrence of NTRK fusions than common cancers like NSCLC (30, 31, 33). In a meta-analysis involving 107 studies, rare cancer types including infantile fibrosarcoma, secretory breast cancer, and congenital mesoblastic nephroma were reported with an incidence of NTRK fusions over 90% (33). However, in other cancer types including NSCLC, nonsecretory breast cancers, pancreatic cancers, renal cell carcinoma, prostate cancer, and melanomas, the frequencies of NTRK fusions were all less than 5%, and most were not up to 1% (30, 33). Furthermore, NTRK fusions were also detected in a large scale of hematologic malignancies with an occurrence of 0.1% in over 7,000 patients, of which a patient with acute myeloid leukemia harboring ETV6-NTRK2 fusion achieved a confirmed response to TRK inhibition therapy (34).

Among the three NTRK genes, NTRK1 and NTRK3 gene fusions can be identified in a wide range of cancer types, NTRK3 fusion is the most common followed by NTRK1 fusion, and ETV6-NTRK3 along with TPM3-NTRK1 are the most common fusion partners (7, 29, 31, 32). NTRK1 fusions are also highly detected in pediatric papillary thyroid carcinomas (32), whereas, ETV6-NTRK3 fusions act as a canonical genetic alteration in secretory carcinoma of salivary glands and breast (24). By contrast, NTRK2 fusions more exclusively exist in central nervous system (CNS) tumors like gliomas, according to a study where NTRK2 fusion was detected in most NTRK fusion-positive patients (9/14) (18, 29). Regarding the co-mutational patterns, NTRK fusions are revealed to barely co-occur with other canonical alterations (7, 35). Previous study revealed that the most frequent co-mutations with NTRK fusions were TP53, PTEN, and PIK3CA mutations, but only one case harbored targetable alterations including EGFR and MET amplification, and 29% (9/31) of patients with NTRK fusion had no other pathogenic alteration (29). Additionally, Rosen et al. described the only one case of 65 cases where NTRK fusion appeared along with activating alterations of classical MAPK pathway oncogenes, yet it later showed a negative expression level of the protein and resistance to larotrectinib (30).

### Frequency and Clinical Characteristics of NTRK Fusions in NSCLC

As for NSCLC patients, the prevalence of NTRK fusions reported in multicontinental studies varies from 0.1% to 3.3%. A meta-analysis mentioned above reported that the frequency of NTRK gene fusions in NSCLC was 0.17% (33). Two large-scale studies showed the frequencies of NTRK gene fusions in NSCLC patients were 0.1% (4/4073) and 0.16%, respectively (29, 30). Another study enrolling 4,872 NSCLC patients estimated an NTRK fusion frequency of 0.23% through NGS (27). In addition, a retrospective study investigating driver gene alterations in 7,395 Chinese NSCLC patients found that the NTRK rearrangement frequency was 0.59% among all patients, 0.61% (33/5378) for patients with lung adenocarcinoma, and 0.5% (4/855) for patients with lung squamous cell carcinoma (36). NTRK fusion was also detected in neuroendocrine carcinoma and sarcomatoid carcinoma of the lung (27, 28, 37). In general, NTRK fusions are far less frequent than other canonical gene fusions in NSCLC, namely ALK, ROS1, and RET (36, 38–40). The common NTRK gene rearrangements in NSCLC were NTRK1 and NTRK3 gene rearrangements (27, 36). Specifically, the occurrence of gene fusions in NSCLC was 0.07%–3.3% for NTRK1 (26, 28, 41), 0.02%–0.2% for NTRK2 (27, 42), and 0.08% for NTRK3 (27).

Consistent with the co-mutation pattern mentioned before, NTRK fusions in NSCLC present a mutually exclusive manner with other canonical mutations and fusions. In a study of 11 NSCLC patients with NRTK fusions, 6 were recognized with co-mutation but none were common oncogenic genes such as KRAS, EGFR, ALK, or ROS1 (27). Evidence can also be found in another study of 91 NSCLC patients, of which the tumor with NTRK1 gene fusions had no known oncogenic alterations (26). The common co-occurrence mutations with NTRK1 fusion were TP53, RB1, and NF1 (28). Although NTRK fusions are reported mostly in middle-aged (a median age of 47.6 years) and non-smoking history populations, which resembles to the clinical profiles of many other fusions, they can also be detected in patients of other age groups or with previous smoking histories, suggesting that NTRK fusions are not related to certain clinical features in NSCLC (27). Furthermore, most NSCLC patients with positive NTRK fusions have metastasis at diagnosis (27). Yet the conclusion is drawn from data of only 11 cases with NTRK gene fusions. Due to the rarity of NTRK gene fusion in NSCLC, studies above were mostly small-scale retrospective studies. Therefore, prospective studies with larger sample size are required to investigate the clinical features of NTRK gene in NSCLC.

## Diagnosis of NTRK Fusions

Generally, nucleic acid-based sequencing is a priority for detecting NTRK fusions, which can be followed by methods like immunohistochemistry (IHC), fluorescence *in situ* hybridization (FISH), and reverse transcriptase polymerase chain reaction (RT-PCR) as complement or substitution when practice environment is limited. Other diagnosis methods have also risen up, such as circulating tumor DNA/RNA testing and nanostring technology. Each method has its own merits and limitations, and some are limited to certain specific clinical conditions. In the following section, we will introduce and compare these techniques individually.

### DNA-Based NGS

NGS shows a great advantage when conducting comprehensive analysis including somatic mutations, insertions, amplifications, deletions, microsatellite instability status, tumor mutation burden, as well as chromosomal rearrangements (43, 44), attributing to its broad capacity of molecular profiling. For example, MSK-IMPACT used in Memorial Sloan Kettering Cancer Center and the FoundationOne CDx test are two broad DNA sequencing panels. Based on hybrid-capture method, the two panels cover the whole coding region of 468 and 324 cancer-related genes, respectively, and are capable of detecting selected fusions including NTRK1, NTRK2, and ETV6-NTRK3 (45, 46). Moreover, high sensitivity and specificity as well as the ability to detect novel fusion partners are advantages of DNA sequencing. Additionally, DNA-based NGS can also function to monitor the development of resistance mutations in patients with NTRK fusions, such as G667C and G595R mutations in NTRK1 gene, and G696A and G623R mutations in NTRK3, which are observed to cause TRK inhibitor resistance (47, 48). However, several technical limitations should be taken into consideration. Practically, the sensitivity is determined by the panel coverage of genomic breakpoints of targeted fusions, and the integrity of its coverage is presented at the breakpoint. Therefore, false negatives could appear because of the limited panel size. In the aforementioned MSK-IMPACT panel, no kinase domain intron of NTRK3 was covered, because the intronic regions of NTRK3 are too long to cover, otherwise the coverage for other genes would be shrunk to reduced overall sensitivity (45). Another reason is that repetitive elements inside some introns are hard to tile and infeasible to assemble (49). Thus, the majority of fusions involving NTRK3 are indirectly detected through identification of the most common fusion partner ETV6, thus the sensitivity is restricted. Furthermore, it is uncertain if novel alterations presented in DNA level can be expressed at the mRNA and protein levels that possess clinical significance (35). Thus, further confirmation by RNA-based sequencing is often necessary. To conclude, broad capacity of molecular profiling, high sensitivity and specificity, and the ability to identify novel fusion partners contribute to the advantages of DNA-based NGS. While limitations of this method include its deficiency to detect NTRK3 fusions, the uncertain RNA-level expression of detected fusions, with the addition of high cost, high sample purity, and long turn-around time.

### RNA-Based NGS

Practically, RNA-based NGS is preferred when it comes to the detection of NTRK fusion. As mentioned, even the most advanced DNA-based sequencing is incapable of covering large intronic regions in NTRK3. However, such limitation does not exist in RNA-based NGS, for introns are already spliced out in RNA. Additionally, sequencing carried out in the RNA level can directly verify in-frame and functionally transcribed genes, which is of potential significance to determine the response to targeted therapy (50). In 232 lung adenocarcinoma samples of which driver alterations were not detected by MSK-IMPACT (DNA sequencing), 36 cases were identified positive for driver alterations through RNA sequencing. Among which, 27 patients were in-frame fusions including two with NTRK3 fusions and one with NTRK2 fusion. Intriguingly, two patients with NTRK fusions receiving larotrectinib treatment achieved confirmed PR or SD (51). Moreover, purity of tumor samples is less required due to the sufficiently high expression of gene fusions. The major disadvantage of RNA-based NGS is the labile nature of RNA extracted from archival samples. In aged materials, the occurrence of RNA fragmentation and degradation is of considerably high probability, which might lead to failure of library preparation and hinder subsequent operations. For instance, a study testing samples of 44 archival cases stated that only 23 cases passed quality control thresholds and were eligible for sequencing (52). Thus, effective quality assessment measures are required to identify potential false-negative results, guaranteeing the test reproducibility (53). Currently, the method termed Anchored multiplex PCR for RNAseq is commercially available and widely applied. In addition to higher sensitivity and specificity, it is effective in detecting single nucleotide variants, copy number variants, insertions, deletions, and gene rearrangements without previous knowledge of the fusion partners (54). It highlights the superiority of RNA-based NGS for NTRK fusion detection to find new fusion partners as well as second resistance in NTRK gene. Thus, RNA-based NGS is preferentially recommended for NTRK fusion detection in tumors where NTRK fusions are uncommon like NSCLC (55). In conclusion, RNA-based NGS can avoid the tough intron issues in the detection of fusions like NTRK3, and is able to directly confirm the transcription of detected fusions, making it an optimal approach for NTRK fusion detection. Yet the unstable RNA quality is a major concern, thus extra labor is required for specimen preservation and quality assessment.

Furthermore, there are some commercially available platforms that are able to simultaneously assess both RNA and DNA. For example, Oncomine Comprehensive Assay by Thermo Fisher and The TruSight Oncology 500 assay by Illumina are hybrid panels including all three NTRK genes (56, 57). Currently, a number of NGS panels based on DNA or RNA are designed for liquid biopsy when no sufficient tumor tissue specimen is available, such as Guardant360 panel (58) and AVENIO Extended ctDNA Analysis Kits (59). However, the sensitivity of such methods still requires future improvement.

### Immunohistochemistry

As a method analyzing protein expression, IHC shows several evident advantages. Primarily, IHC is widely used in laboratories, due to its relatively low expense and low implementation threshold with only one single unstained slide and approximately a day of turnaround time. Moreover, IHC presents higher confidence that fusions detected are functionally transcribed and translated, allowing a spatial assessment of the subcellular localization of the fusion protein, which is indicative for oncogenic activity and targeted therapy. In addition, IHC presents high sensitivity and specificity (29, 35, 60, 61). EPR17341 (Abcam, Cambridge, MA, USA), a pan-TRK monoclonal antibody, is mostly used and is able to detect proteins TRKA, TRKB, and TRKC expression (35). However, the utility of IHC is restricted in diagnosis of NTRK fusions. Initially, the exact fusion partners and precise breakpoints cannot be identified, since only TRKs are targeted. Second, false positivity may occur as TRK proteins are not only specific to NTRK fusions. For instance, TRK proteins can also be expressed in normal tissues and tumor tissues with neuronal and smooth muscle differentiation, which do not harbor valid fusions, while the specificity was high for lung cancer (45, 61). Furthermore, sensitivity decrease of IHC for TRKC was revealed. Zoran et al. reported the sensitivity as 55% (29), while Solomon et al. have found a sensitivity of 79% for NTRK3 fusions, in contrast with the sensitivity of 96% and 100% for NTRK1 and NTRK2 fusions, respectively (45). Moreover, there are no monoclonal TRKC antibodies commercially available, thus, identification specific to NTRK3 fusions remains stagnated. Finally, the present estimated sensitivity and specificity data are established on research of small samples with NTRK fusion positive, suggesting that verification from studies with larger cohorts is required. Overall, IHC is a convenient, economic, and effective testing method. The detected fusion proteins could provide significant indications for clinical treatment. However, its incapability to identify fusion partners, ineffectiveness to detect TRKC, and false positive results due to the non-specific expression of TRKs jointly limit the application of IHC. Therefore, IHC mainly perform as a screening tool for NTRK fusion when NGS is not available or serve as an adjunct to nucleic acid testing, but orthogonal confirmation through NGS should be conducted for higher sensitivity if possible.

### Fluorescence *In Situ* Hybridization

FISH is extensively used for detecting oncogenic fusions in solid tumors *via* chromosomal rearrangement analysis. In addition to the good sensitivity and specificity, it requires only one or two slides and lower tumor purity and takes only a few-day turnaround time. Notably, FISH is highly effective for identifying ETV6-NTRK3 fusions, which enables its good application in mammary analog secretory carcinoma, infantile fibrosarcoma, and congenital mesoblastic nephroma (52, 62). A break-apart probe (Abbott, Chicago, IL) is used specifically for the detection of ETV6 gene. There are also break-apart probes targeting the three NTRK genes and are commercially available (63, 64). Still, there are demerits in NTRK fusion detection. First, three FISH assays are required to be performed to assess three NTRK genes (65), which consequently costs more expense and time. Second, FISH is unable to ascertain the 5’ partner of the fusion, while NTRK fusions involve multiple partners of great clinical significance. Third, higher probability of false-negative results is presented particularly for NTRK1 fusions. According to a study of short inversions and intrachromosomal translocations related to ALK, split lengths separated by the break-apart probe is too short to be distinguished from normal types (66). Given that most NTRK1 fusions are formed in a intrachromosomal manner, false-negative results could appear by insufficient splitting of FISH (67). Finally, no certainty could be made in FISH that the fusion detected on the DNA level can be functionally transcribed and finally translated. In brief, FISH is a widely-applied fusion-testing approach with credible sensitivity and specificity, and particularly serves as a potent tool for ETV6-NTRK3 fusion detection. Nevertheless, it fails to recognize fusion partners, and its sensitivity for NTRK1 is questionable.

### RT-PCR

Reverse transcriptase polymerase chain reaction is a method based on the detection of transcribed RNA, in which either qualitative assay or quantitative real-time PCR could be performed. As fusion partners and corresponding exon breakpoints both required clarification before an RT-PCR assay can be conducted, noncanonical and novel fusions could not be identified. In the past years, it has been used mainly for detecting canonical ETV6-NTRK3 fusions, thus its applicability is limited to cases enriched of such alterations (64, 68, 69). However, its sensibility needs further evaluation. In a study involving 25 cases of salivary gland secretory carcinoma which were proven to be canonical fusion negative *via* RT-PCR, four cases of which were found harboring classical fusion through more sensitive nested RT-PCR, and five atypical ETV6 exon4-NTRK3 exon 14 or ETV6 exon5-NTRK3 exon14 fusions were identified by both PCR and nested RT-PCR (64), which suggests a considerable possibility of false-negative results. To conclude, RT-PCR can perform well in ETV6-NTRK3 fusion detection, but its sensibility still requires improvement. Besides, recognition of non-canonical and novel fusions is beyond its category. Therefore, the utility of RT-PCR is largely limited by the highly variable fusion partners, exons, and breakpoints involved in NTRK fusions.

## TRK Inhibitors and Resistance

The first-generation NTRK-TKIs (larotrectinib and entrectinib) have demonstrated clinically meaningful antitumor activity (**Table 1**), thus had been approved for the treatment of locally advanced or metastatic patients with NTRK-rearranged solid tumors. According to the NCCN guidelines, both larotrectinib and entrectinib are recommended as standard therapies for the first-line treatment of NTRK fusion-positive patients with advanced or metastatic NSCLC, as well as progressive patients with previous systemic therapies. However, primary or acquired resistance to first-generation NTRK-TKIs is inevitable. The mechanisms of acquired resistance include “on-target” mechanisms, secondary mutations occurring at the TRK kinase domain, and “off-target” mechanisms, such as bypass signaling pathways activation (48, 70, 71). However, the mechanisms of primary resistance remain unclear. Currently, the mechanism of resistance to TRK inhibitors and next-generation TRK inhibitors are under development, and ongoing clinical trials are in search of appropriate therapeutic strategies (**Table 2**).

**Table 1:** The efficacy of the first-generation TRK inhibitors.

TRK inhibitor	Overall population					NSCLC		
	*N*	ORR	PFS	CNS ORR	CNS PFS	*N*	ORR	CNS ORR
Larotrectinib	159	79% (121/153)	28.3 (22.1–NE)	75% (9/12)	NA	12	75% (9/12)	NA
Entrectinib	54	57% (31/54)	11.2 (8.0–14.9)	50% (6/12)	7.7 (4.7–NE)	10	70% (7/10)	NA

NE, not estimable; NA, not available.

**Table 2:** Ongoing clinical trials for NTRK fusion-positive tumor.

ID	Drug	Phase	Gene fusion	Tumor type	Age	Primary outcome measures	Status
NCT02576431	Larotrectinib	Phase 2	NTRK	Solid tumors	18 Years and older	ORR	Recruiting
NCT04671849	SIM1803-1A	Phase 1	NTRK, ROS1, ALK	Solid tumors	18 Years and older	AEs, dose expansion	Recruiting
NCT03215511	Selitrectinib	Phase 1/2	NTRK	Solid tumors	1 Month and older	Phase 1: recommended dose, MTD	Active, not recruiting
						Phase 2: ORR	
NCT04687423	FCN-011	Phase 1/2	NTRK	Solid tumors	16 Years and older	TRAEs, RP2D, ORR	Recruiting
NCT04996121	XZP-5955	Phase 1/2	NTRK, ROS1	Solid tumors	18 years and older	MTD, AEs, ORR	Not yet recruiting
NCT04094610	Repotrectinib	Phase 1/2	NTRK, ROS1, ALK	Solid tumors, lymphoma	Up to 25 years	Phase 1: DLTs, RP2D	Recruiting
						Phase 2: ORR	
NCT04617054	AB-106	Phase 2	NTRK	Solid tumors	18 Years and older	BOR	Recruiting
NCT01639508	Cabozantinib	Phase 2	RET, ROS1, NTRK	NSCLC	18 Years and older	ORR	Recruiting
NCT04901806	PBI-200	Phase 1/2	NTRK	Solid tumors	18 Years and older	Phase 1: AEs, RP2D	Recruiting
						Phase 2: ORR	
NCT02920996	Merestinib	Phase 2	NTRK	Solid tumors	18 Years and older	ORR	Active, not recruiting
NCT02675491	DS-6051b	Phase 1	NTRK, ROS1	Solid tumors	20 Years and older	AEs	Active, not recruiting
NCT03556228^a^	VMD-928	Phase 1	NTRK1	Solid tumors, lymphoma	18 Years and older	AEs	Recruiting
NCT02637687 (SCOUT)	Larotrectinib	Phase 1/2	NTRK	Solid tumors	Up to 21 years	Phase 1: TEAEs, DLT	Recruiting
						Phase 2: ORR	
NCT02568267 (STARTRK-2)	Entrectinib	Phase 2	NTRK, ROS1, ALK	Solid tumors	18 Years and older	ORR	Recruiting
NCT03093116 (TRIDENT-1)	Repotrectinib	Phase 1/2	NTRK, ROS1, ALK	Solid tumors	12 Years and older	Phase 1: DLTs, RP2D	Recruiting
						Phase 2: ORR	
NCT04655404	Larotrectinib	Early phase 1	NTRK	High-grade glioma	Up to 21 years	DCR, TEAEs, AUC, dose–response relationship	Recruiting
NCT03213704	Larotrectinib	Phase 2	NTRK	Solid tumors, non-Hodgkin lymphoma	12 Months to 21 years	ORR	Recruiting
NCT04302025	Entrectinib	Phase 2	ROS1N, TRK	NSCLC	18 Years and older	MPR	Recruiting
NCT03994796	Entrectinib	Phase 2	NTRK, ROS1	Solid rumors with BM	18 Years and older	ORR	Recruiting
NCT03834961	Larotrectinib	Phase 2	NTRK	Solid tumors, acute leukemia	Up to 30 years	ORR	Recruiting
NCT02650401 (STARTRK-NG)	Entrectinib	Phase 1/2	NTRK, ROS1	Solid tumors	Up to 18 years	MTD, RP2D, ORR	Recruiting
NCT02465060	Larotrectinib	Phase 2	NTRK	Solid tumors, lymphoma, multiple myeloma	18 Years and older	ORR	Recruiting

Inclusion criteria also include NTRK1 gene amplifications or TRKA protein overexpression.

AEs, adverse events; AUC, area under the curve; BM, brain metastases; BOR, best overall response; DCR, disease control rate; DLT, dose-limiting toxicity; MPR, major pathologic response; MTD, maximum tolerated dose; ORR, overall response rate; RP2D, recommended phase 2 dose; TEAEs, treatment emergent adverse events; TRAEs, treatment-related adverse events.

### First-Generation TRK Inhibitors

Larotrectinib, an oral small-molecule and highly selective pan-TRK inhibitor, was initially approved for adults and pediatric patients with locally advanced or metastatic solid tumors harboring NTRK gene fusions without known acquired resistance mutations in the USA in November 2018 (72), as the first tissue-agnostic nod of targeted therapy. The antitumor activity of larotrectinib in patients with locally advanced or metastatic solid tumors harboring NTRK fusions has been explored in three clinical trials, including a phase I adult trial (NCT02122913) (73), a phase I/II pediatric trial (SCOUT, NCT02637687) (74), and a phase II adult and adolescent trial (NAVIGATE, NCT02576431). The phase I dose-escalation study of larotrectinib (NCT02122913) recruited 8 patients with NTRK gene fusions; the overall response rate (ORR) was 100% by independent review, including 2 patients assessed as complete responses (CR) and 6 patients assessed as partial responses (PR) (73). Drilon et al. reported the results of a primary analysis set of 55 patients with TRK fusion-positive solid tumors in 3 trials (NCT02122913, NCT02637687, and NCT02576431). The ORR was 75% (95% CI, 61–85) according to the independent review committee and 80% (95% CI, 67–90) determined by the investigator’s assessment (47). Thus, the approval of larotrectinib was based on which. Hong et al. reported the pooled analysis result of the abovementioned three phase I/II clinical trials of larotrectinib (**Table 1**) (75). The ORR was 79% (121/153), the median progression-free survival (PFS) was 28.3 months (95% CI 22.1–NE), and the median overall survival (OS) was 44.4 months (95% CI, 36.5–NE) in the overall population. In the subgroup of NSCLC, the ORR was 75% (9/12). Furthermore, the efficacy of larotrectinib was independent of the NTRK gene. There were 13 (8%) of 159 patients with brain metastases, and a response to larotrectinib was observed in 9 of 12 (75%) of these patients. In patients who received larotrectinib treatment with 0, 1–2, and more than 3 prior lines of therapy, the ORR was 86%, 63%, and 80%, respectively, the median duration of response (DOR) was 27.6 months, not reached, and 32.9 months, respectively, and the median PFS was 29.4, 33.4, and 34.5 months, respectively, suggesting that the efficacy of larotrectinib is independent of prior treatments (76). In addition, a retrospective analysis showed that larotrectinib can improve PFS for previous treated patients with advanced TRK fusion cancer (77). There are several recruiting clinical trials that tend to further explore the efficacy of the larotrectinib in patients with NTRK fusion, and the tumor types of patients enrolled included acute leukemia, lymphoma, or central nervous system neoplasm (NCT03834961, NCT04655404, NCT03213704, NCT02465060). Interestingly, two cases harboring NTRK1 gene amplification were reported a partial response after treatment with larotrectinib, which indicated that larotrectinib may be effective for patients with NTRK gene amplification as well as NTRK fusions (73, 78). Moreover, there are clinical trials (NCT04879121, NCT02693535) exploring the effect of larotrectinib for patients with locally advanced or metastatic solid tumors harboring NTRK amplification. Adverse events of larotrectinib were predominantly of grade 1 or 2, with the most common adverse events being anemia, an increase in the alanine aminotransferase or aspartate aminotransferase level, and a decrease in the neutrophil count (47, 73, 75). Improvement in health-related quality of life was also observed after treatment with larotrectinib (79).

Entrectinib, an oral selective inhibitor of TRKA/B/C, ROS1, and ALK tyrosine kinases, received its first approval for the treatment of advanced or recurrent adult and pediatric solid tumors with positive NTRK fusion in Japan in June 2019 (80). Then, entrectinib soon received approval for such indication by the FDA in August 2019 (81). It has also been approved for the treatment of adult patients with advanced ROS1 fusion-positive NSCLC. The safety and efficacy of entrectinib have been explored in four clinical trials: a phase I trial ALKA-372-001, a phase I trial in adults (STARTRK-1, NCT02097810), a phase I/II study in children and adolescents (STARTRK-NG, NCT02650401), and a phase II basket trial in adults (STARTRK-2, NCT02568267). Doebele et al. reported an integrated analysis results of three phases I–II trials (ALKA-372-001, STARTRK-1, STARTRK-2) that evaluated entrectinib in patients with advanced or metastatic solid tumors with fusion-positive NTRK (**Table 1**) (82). In the efficacy-evaluable population, the ORR was 57% (31/54) and the median PFS and OS were 11.2 (8.0–14.9) and 21 (14.9–not estimable) months, respectively. In patients with baseline CNS metastatic, the ORR was 50% (6/12) and the median PFS was 7.7 (4.7–not estimable) months. In the subgroup of NSCLC, the ORR was 70% (7/10). Furthermore, intrapatient comparisons of entrectinib efficacy in the STARTRK-2 trial indicated that the ORR was higher and the median PFS was longer for entrectinib than discontinuation since the last therapy (83). Additionally, a case report showed that a patient with SQSTM1-NTRK1 fusion-positive advanced lung adenocarcinoma was treated with entrectinib, then developed partial response and had a complete remission of all brain metastases (41). In summary, treatment with entrectinib led to clinically significant antitumor activity in patients with positive NTRK fusion. Importantly, entrectinib is also effective for CNS tumors or CNS metastases. This is likely due to sustaining CNS exposure of entrectinib, because it is a weak p-glycoprotein substrate different from crizotinib and larotrectinib which are strong p-glycoprotein substrates with poor brain penetration (84). Currently, a head-to-head study comparing the efficacy of entrectinib and crizotinib in patients with advanced or metastatic ROS1+ NSCLC with and without CNS metastases is recruiting (NCT04603807). As for the safety analysis, most adverse events are grade 1 or 2 and reversible, and the common treatment-related adverse events include dysgeusia, fatigue, dizziness, constipation, etc. The commonly reported grade 3 or 4 adverse events are increased weight and anemia, while cognitive disorder is the most common serious treatment-related event (82). Thus, we conclude that entrectinib is an effective therapy with minor adverse events for advanced patients with NTRK gene fusions, including patients with primary CNS tumors and metastatic CNS diseases. Meaningfully, entrectinib as neoadjuvant therapy in patients with resectable stages II–III NSCLC is currently under investigation (NCT04302025), and the results of which may provide a novel perspective for therapeutic strategies in NSCLC.

### First-Generation TRK Inhibitor Resistance

#### “On-Target” Mechanisms

**Figure 2:**
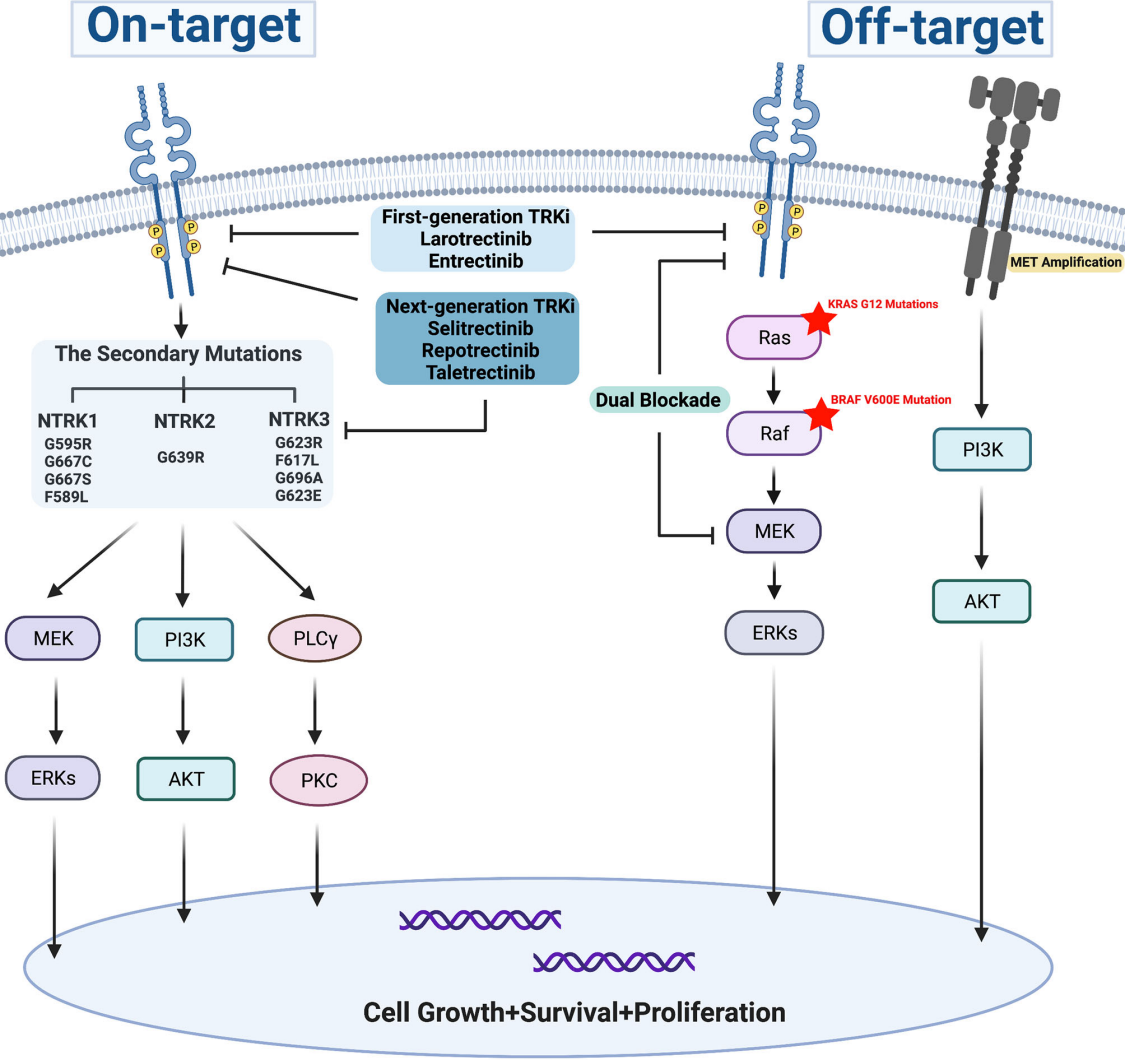
Resistance mechanism for first-generation TRK inhibitors.

The secondary mutations occurring at the ATP binding pocket of the TRK kinase domain includes the solvent-front, gatekeeper region, and xDFG motif mutations in the activation loop, also known as ‘on-target’ mechanisms, which represent the common acquired-resistance mechanisms for the first-generation TRK inhibitors (**Figure 2**). Up to now, several resistance mutations have been reported. In 2015, the solvent-front mutations (G595R) and xDFG motif mutation (G667C) in the TRKA kinase domain were initially reported as acquired resistance mechanisms to entrectinib in a patient with colorectal cancer involving LMNA-TRKA rearrangement (48). Then, a NTRK3 G623R mutation was reported to be related to acquired resistance to entrectinib in a patient with mammary analog secretory carcinoma with ETV6-NTRK3 fusion (25). Later, a novel gatekeeper region (F589L) mutation in TRKA, the xDFG mutations (NTRK1 G667S, NTRK3 G696A), and solvent front mutations (NTRK1 G595R, NTRK3 G623R) were identified as resistance mechanisms to larotrectinib (47). Furthermore, NTRK1 G595R and NTRK1 G667S mutations presented in a NSCLC patient, and a gatekeeper mutation (NTRK3 F617L) presented in a patient with gastrointestinal stromal tumor after disease progression with larotrectinib treatment (73). On-target secondary resistant mutations bring about amino acid substitutions, thus result in sterically preventing the binding of the first-generation TRK inhibitors. Next-generation TRK inhibitors have already been developed to overcome the on-target resistance mutations during treatment with first-generation TRK inhibitors.

#### “Off-Target” Mechanisms

Off-target mechanisms can develop during TRK inhibitor treatment, which include genomic alterations of downstream pathway mediators and other receptor tyrosine kinases (**Figure 2**). Preclinical study showed that the reactivation of RAF-MEK-ERK signaling was observed in NTRK1-driven pancreatic cancer and lung cancer treated with entrectinib, which was possibly one of the acquired-resistance mechanisms to entrectinib, and combined inhibition of TRKA plus MEK1/2 markedly forestalled the onset of drug resistance in both models (85). Furthermore, BRAF V600E mutation, KRAS G12D mutations, and MET amplifications were also identified as the bypass-mediated resistance mechanisms to TRK inhibitors for patients with NTRK fusions. Dual blockade of TRK and MEK could effectively control tumor growth and delay the emergence of off-target resistance (71). However, the next-generation TRK inhibitor monotherapy was not effective for resistance mediated by bypass pathway mutations (71, 86, 87). In a case of pancreatic adenocarcinoma with CTRC-NTRK1 gene fusion, BRAF-V600E mutation emerged when disease progressed with larotrectinib, which previously achieved a PR at its best, then the tumor continued to progress for 2 months even though the treatment was switched to selitrectinib, a next-generation TRK inhibitor (86). Intriguingly, these data provide clues for combination therapies of blocking both NTRK and MEK in NTRK fusion-positive tumors for future investigations.

### Next-Generation TRK Inhibitors

Selitrectinib (LOXO-195), a selective TRK inhibitor, was designed to overcome acquired resistance to first-generation TRK inhibitors mediated by secondary mutations in kinase domain. LOXO-195 showed significant inhibitory cellular activity against NTRK fusions and acquired resistance mutations *in vitro*, including TRKA G595R, TRKA G667C, and TRKC G623R (88). Notably, LOXO-195 possessed antitumor activity in two patients that had LMNA-NTRK1 fusion-positive colorectal cancer and ETV6-NTRK3 fusion-positive infantile fibrosarcoma with TRKA G595R- and TRKC G623R-driven acquired resistance to larotrectinib, respectively (88). Furthermore, selitrectinib response was also observed in a patient with NTRK3 G623R mutation and CNS metastasis who has acquired resistance to entrectinib with ETV6-NTRK3 fusion-positive mammary analog secretory carcinoma of the parotid gland (89). In a phase I/II study (NCT03215511, *n* = 20) and FDA-expanded access single patient protocol (SPP, *n* = 11), the ORR of LOXO-195 was 34% (10/29) in all evaluable patients, and the ORR was 45% (9/20) in patients with TRK kinase mutation, but the ORR was 0% (0/3) in patients with resistance mediated by identified bypass, and the most common adverse events were dizziness/ataxia, nausea/vomiting, anemia, myalgia, abdominal pain, fatigue, and lymphopenia (87). It suggests that LOXO-195 is significantly effective in patients with resistance to prior TRK inhibitors mediated by mutations in kinase domain but not bypass pathway activation. However, LOXO-195 exhibited limited response to a pediatric glioma driven by ETV6-NTRK3 fusion with G623A- and G623E-resistant mutations. It was possibly due to the insufficient CNS concentrations of LOXO-195 and trophic microenvironment of the pediatric brain that confers resistance to TRK inhibitors (90). LOXO-195 possessed poor penetration into the brain because of the blood–brain barrier and multidrug efflux transporters, such as ABCB1 and ABCG2 (91, 92). In addition, clinical evidences and preclinical findings revealed that TRKA xDFG motif substitutions, such as TRKA G667A and TRKA G667C, conferred resistance to the next-generation TRK inhibitors including selitrectinib and repotrectinib through impaired drug binding (93). Recently, a case report showed that a patient with DCTN1-NTRK1 fusion-positive undifferentiated pleomorphic sarcoma did not respond to LOXO-195 who harbored acquired NTRK1 G667C mutation after disease progression with larotrectinib (94). Thus, resistance mediated by xDFG mutation remains a major challenge for next-generation TRK inhibitors. Though recent studies report that promising drug compounds designed to overcome multiple resistance possessed potent inhibitory activities to xDFG mutations as well as solvent-front and gatekeeper substitutions *in vitro* and *in vivo* (95, 96), the exploration of new drugs to inhibit xDFG mutation is still facing unmet clinical needs.

Repotrectinib (TPX-0005) is a novel next-generation ALK, ROS1, and pan-TRK inhibitor, which is designed to overcome resistance mutations and potently inhibit wildtype TRK fusions. Repotrectinib is highly potent and selective against wildtype ALK, ROS1, and TRK fusion proteins, as well as their solvent-front substitutions in preclinical studies, including TRKA G595R, TRKB G639R, and TRKC G623R (97, 98). Similarly, a dramatic response to repotrectinib was observed in a patient with NTRK3 fusion-positive mammary analog secretory carcinoma harboring NTRK3 G623E mutation. Notably, repotrectinib achieved partial response in NSCLC patients with ROS1 fusion and intracranial metastasis, who were treatment naive or presented solvent-front mutation-mediated resistance to previous ROS1-TKI, demonstrating an efficient intracranial antitumor activity of repotrectinib (97, 99). Efficient CNS penetration of repotrectinib was observed in patients and mouse models, but inconsistent result was showed in a bioanalytical assay, revealing that repotrectinib possessed very poor penetration into the brain in mouse experiment, probably because of the blood–brain barrier and multidrug efflux transporters, like ABCB1 and ABCG2 (100, 101). The potent intracranial activity of repotrectinib in patients with NTRK-fusion tumors including NSCLC remains unclear, requiring further investigation. A clinical trial of repotrectinib in patients with advanced solid tumors harboring NTRK, ALK, or ROS1 rearrangements (TRIDENT-1, NCT03093116) are currently being conducted, of which the interim data showed evident antitumor activity of repotrectinib in patients harboring NTRK fusion-positive cancers both with and without previous NTRK-TKI treatment (98). Two cases of metastatic NSCLC harboring NTRK3 rearrangement from TRIDENT-1 study achieved durable responses to repotrectinib, with one being NTRK-TKI naive and one with previous entrectinib resistance mediated by G623R mutation (82). What is more, repotrectinib was more potent against wildtype TRK fusions and mutations in TRK kinase domain than selitrectinib in cellular assays and mouse models. Repotrectinib was also the only TRK inhibitor active against TRKA G595R/F589L compound mutation in cis in preclinical Ba/F3 cells (102). This indicates that repotrectinib is more efficient for wildtype TRK fusions and secondary resistance mutations in preclinical studies, though evidence from clinical study is still insufficient. Currently, phase I/II clinical trials (NCT03093116, NCT04094610) are ongoing to explore the efficiency of repotrectinib in patients with advanced solid tumors harboring NTRK, ROS1, and ALK rearrangements.

Taletrectinib (DS-6051b/AB-106) is a selective tyrosine kinase inhibitor of NTRK and ROS1. Preclinical study showed that DS-6051b was significantly effective in inhibiting NTRK and ROS1-rearranged cancers, as well as TKI-resistant tumors with secondary kinase domain mutations, such as G2032R mutation in ROS1 and G595R mutation in NTRK1 (103). However, NTRK1 G667C mutation was resistant to DS-6051b; it was consistent with previous reports claiming G667C mutation in xDFG motif being resistant to next-generation TRK inhibitors (93, 103). Preliminary clinical activity of DS-6051b was observed in TKI-naive and crizotinib-pretreated ROS1+ NSCLC patients and a patient with TPM3-NTRK fusion-positive thyroid cancer who achieved a confirmed partial response of 27 months at the last follow-up (104, 105). The evidence about antitumor effect of taletrectinib in patients with advanced NSCLC harboring NTRK fusion is insufficient; thus, further investigation is required. The most common treatment-related adverse events are elevation of aspartate aminotransferase and alanine aminotransferase, nausea, diarrhea, and vomiting (104, 106).

### Next-Generation TRK Inhibitor Resistance

As stated above, resistance mechanisms of tyrosine kinase inhibitors typically include on-target and off-target mechanisms, while resistance mechanisms of next-generation TRK inhibitors are yet to be well described. Two patients, one with TPR-NTRK1-positive NSCLC and the other one with TPM3-NTRK1-positive thyroid cancer, harboring xDFG motif mutations (TRKA G667C, G667S) that emerged as resistance to larotrectinib, did not respond to next-generation TRK inhibitor selitrectinib, which represented one of the primary resistance mechanisms to next-generation TRK inhibitor (93). Furthermore, patients achieved partial response to selitrectinib against TRKA G595R-mediated larotrectinib resistance, while TRKA G667C or TRKA G667A were detected at progression during selitrectinib treatment, indicating that TRKA G667 mutations were responsible for acquired resistance to next-generation TRK inhibitors (93). Consistent results were observed in preclinical models, where NTRK1 G667 mutation was found insensitive to next-generation TRK inhibitors, including selitrectinib, repotrectinib, and DS-6051b (93, 103). Importantly, xDFG motif mutations (NTRK1 G667) were highly sensitive to type II inhibitors, including altiratinib, cabozantinib, and foretinib in preclinical studies (93, 107). Also, foretinib and nintedanib significantly inhibited the growth of cells with TRKA G667C mutation, and foretinib was also effective against NTRK1-G667C mutation in a brain metastasis model (108). Moreover, Ba/F3 cells expressing TPM3-NTRK1 G667C or TPM3-NTRK1 fusion were sensitive to gilteritinib but it failed to suppress G595R-mutant cells (109). This calls on further studies to overcome G667 mutations. Additionally, in a case with metastatic undifferentiated sarcoma harboring TPM3-NTRK1 fusion, selitrectinib was used to overcome acquired resistance to larotrectinib with a secondary G595R mutation. KRAS G12V mutation and functional activation of KRAS signaling were later identified in the lesion developing resistance to selitrectinib (110). Similarly, a patient with colorectal cancer harboring LMNA-NTRK1 fusion showed emergence of KRAS G12A and G12D mutations when developing acquired resistance to LOXO-195 (71). This indicated that bypass pathway activating *via* KRAS mutations was one of the resistance mechanisms to selitrectinib, and further exploration of other mechanisms is urgently needed for appropriate therapeutic strategies toward resistance to next-generation TRK inhibitors.

## NTRK Fusion With EGFR-TKI Resistance

Interestingly, NTRK fusions are recognized as a resistance mechanism to EGFR-TKIs in NSCLC patients (28, 111). According to a survey investigating 3,050 EGFR+ NSCLC samples, the emergence of TPM3-NTRK1 was confirmed to follow the initiation of EGFR-TKI erlotinib treatment through the comparison between paired pre- and after-treatment samples (111). Consistent results can also be seen in other studies, where TPM3-NTRK1 fusion was detected in patients with resistance to third-generation EGFR-TKI osimertinib or rociletinib (112, 113). Notably, in a large-scale cohort involving Chinese lung cancer patients, six of twelve patients with NTRK1 fusion-positive NSCLC had co-occurring EGFR mutations and were previously treated with EGFR-TKIs, suggesting that NTRK1 fusions were the potential resistance mechanisms to EGFR-TKIs regardless of its generation (28). A NSCLC patient with EGFR 19del received gefitinib followed by osimertinib because of the emergence of EGFR T790M, then EGFR C797S and LMNA-NTRK1 fusion were detected when resisting to osimertinib. Notably, the patient showed continuous slow disease progression for 9 months with osimertinib combined with crizotinib as an TRK inhibitor (28). Moreover, a patient with IRF2BP2-NTRK1 lung adenocarcinoma achieved a durable stable disease to crizotinib for 16 months (114). It revealed the antitumor effect of crizotinib for NTRK fusion-positive NSCLC, suggesting that combining EGFR-TKIs and TRK inhibitors may be an optional treatment for patients with NTRK fusion-mediated EGFR-TKI resistance. The effect of first- and next-generation TRK inhibitors for EGFR-TKI-resistant tumors with NRTK fusions requires further investigation for better comprehension of resistance mechanism.

## NTRK Fusion and Immunotherapy

In recent years, immune checkpoint inhibitors (ICIs) have remarkably changed the treatment landscape of cancers like NSCLC. However, the clinical efficacy and safety of ICIs for patients with NTRK fusion positive remains unknown. There are several studies exploring the relationship between NTRK fusion and biomarkers for ICIs, including PD-L1 expression, microsatellite instability, and tumor mutation burden (TMB), which had been identified as predictive biomarkers for ICIs (115–117). Evidence can be found in 31 cases with NTRK fusions, where PD-L1 expression was detected in 23% of cases with NTRK fusions, but only 2 cases possessed high microsatellite instability (MSI-H) (29). With the exception of colorectal cancer, NTRK fusions was demonstrated to be positively related to MSI-H and mismatch repair deficiency (MMR-D) (7, 118, 119). A study showed that 6 of 7 patients with NTRK fusion-positive colorectal cancers were MSI-H and possessed high median TMB. This is consistent with another finding that of 12 patients with NTRK fusions including two MSI-H colorectal cancers, only a patient with colorectal cancer achieved a complete response to ICIs (30). Additionally, NTRK fusion-positive tumors presented a lower TMB than those with NTRK fusion negative, excluding MSI-H colorectal cancers, which may be due to the uncommon appearance of NTRK fusion co-existing with alternative oncogenic drivers (30). As for lung cancer, previous studies revealed that it had a significantly higher median TMB but a lower frequency of MSI-H compared with other solid tumors (115, 116). However, the association between NTRK fusion and TMB is still unclear in NSCLC. Results from a large real-world study revealed that the median TMB was similar in NTRK fusion-positive and fusion-negative NSCLC. Additionally, a genomic testing of 2,522 lung adenocarcinomas showed that gene fusion was significantly enriched in driver-negative samples with low TMB, the median TMB for fusion-positive and fusion-negative samples were 1.97 and 5.58 mutations/Mb, respectively, yet the analysis was based on all fusion-positive samples and not specific to NTRK fusion (51). As for immunotherapy, a patient with lung adenocarcinoma harboring NTRK fusion receiving anti-PD1/PDL1 treatment achieved stable disease (30). However, inconsistent result emerged in a case report, where a patient with advanced lung adenocarcinoma harboring a novel NCOR2-NTRK1 fusion showed disease progression after receiving two cycles of anti-PD-1 inhibitor monotherapy, although the presence of high TMB (58.58 mutations/Mb) and positive PD-L1 expression (20%–30% of the tumor cells) was also observed in this case. Predominantly, the patient showed a partial response after switching to TRK inhibitor larotrectinib (120). It indicates that TRK inhibitor is more effective than anti-PD-1 inhibitor monotherapy for patients with NTRK fusion-positive NSCLC in spite of higher TMB and positive PD-L1 expression simultaneously. However, there is no sufficient evidence to draw conclusions based on this single case report. Regarding the efficacy comparison of TRK inhibitors and ICIs, further investigations are required. Whether TRK inhibitors combining with PD-1/PD-L1 inhibitors have superior performance than monotherapy is also worthy of exploration.

## Conclusion

NTRK gene fusions are identified as oncogenic drivers of various adult and pediatric solid tumors, and the prevalence of NTRK fusions varies by tumor types. In NSCLC, NTRK fusions are rare, with an overall prevalence of below 5% and mostly less than 1%. No clear evidence has been linking NTRK fusion to certain clinical features, but it has been revealed that NTRK fusion is mutually exclusive with other canonical mutations. The first-generation TRK inhibitors (larotrectinib and entrectinib) showed remarkable efficacy and good safety for locally advanced or metastatic patients with NTRK fusions, thus they had been approved for the treatment of NTRK fusion-positive solid tumors by the FDA. However, resistance is developed inevitably, and the typical mechanisms of resistance to first-generation TRK inhibitors include secondary mutations in TRK kinase domain and bypass signaling activation. Subsequently, next-generation TRK inhibitors (selitrectinib, repotrectinib, and taletrectinib) are designed to overcome acquired resistance mediated by secondary mutations in TRK kinase domain, which are predominant against wildtype TRK and secondary mutations. Previous studies have revealed that xDFG motif substitutions in TRK induce resistance to next-generation TRK inhibitors, but are high sensitivity to type II inhibitors, which highlights areas for future study. Interestingly, NTRK fusion was reported as a potential resistance mechanism to EGFR-TKIs, suggesting that combining EGFR-TKIs with TRK inhibitors may be an optional treatment for patient with NTRK fusion-mediated EGFR-TKI resistance. Thus, it indicates the importance of detecting NTRK fusions, secondary mutations, and bypass signaling in patients with NSCLC, which provides clues for appropriate therapeutic strategies. Also, the RNA-based NGS is preferentially recommended for NTRK detection in tumors including NSCLC. In terms of immunotherapy, no response was observed in two cases with NSCLC, the efficacy of ICIs in patients with NTRK fusion has not been well described, and whether combination of TRK inhibitors with ICIs possesses better efficacy and safety is not yet clear, thus, further investigation is urgently required to address these issues more fully.

## Author Contributions

FL carried out the primary literature search and drafted and revised the manuscript. YW and HZ contributed to drafting and revising of the manuscript. JJ and PZ helped modify the manuscript. JJ and QC carried out the literature analysis and revised the manuscript. All authors read and approved the final manuscript.

## Funding

This work was supported by the National Natural Science Foundation of China (Grant Nos. 82072597, 62131009, and 81974483) and Chinese Society of Clinical Oncology Research Funding (Y-XD2019-214 and Y-2019Roche-149).

## Conflict of Interest

The authors declare that the research was conducted in the absence of any commercial or financial relationships that could be construed as a potential conflict of interest.

## Publisher’s Note

All claims expressed in this article are solely those of the authors and do not necessarily represent those of their affiliated organizations, or those of the publisher, the editors and the reviewers. Any product that may be evaluated in this article, or claim that may be made by its manufacturer, is not guaranteed or endorsed by the publisher.
